# Early Versus Delayed Double J Stent Removal in Deceased Donor Renal Transplant Recipients: A Prospective Comparative Study

**DOI:** 10.7759/cureus.3006

**Published:** 2018-07-19

**Authors:** Balaji Appiya Ramamoorthy, Prakash Javangula Venkata Surya, Danny Darlington

**Affiliations:** 1 Urology, Government Stanley Medical College And Hospital, Chennai, IND

**Keywords:** deceased donor renal transplantation, double j stent, early removal, urinary tract infections

## Abstract

Introduction: Prophylactic ureteric stents have been commonly employed to reduce the incidence of ureteric obstruction and anastomotic leaks following renal transplantation. There are very few studies on the duration of ureteric stenting in deceased donor renal transplantation (DDRT). We performed a prospective study to compare early and late double J stent removal in DDRT.

Aims and methods: The aim of this study was to compare the early and delayed removal of ureteric stents after DDRT. We performed 80 DDRTs at our center from August 2012 to December 2016, which were included in the study. However, we enrolled 48 patients as the remaining had to be excluded based on the high-risk factors. The recipients were allocated on the 14th postoperative day to two groups. Group 1 underwent early stent removal on the fourteenth postoperative day and in group 2, the stent was removed in the sixth postoperative week. The two groups were followed up for six months and the incidence of urological complications and urinary tract infections (UTI) was compared.

Results: The incidence of UTI during the follow-up period of six months was significantly lower in the early stent removal group (two out of 24) than in delayed stent removal group (eight out of 24) (p=0.016). Asymptomatic bacteriuria occurred in six out of 24 (25%) in group 1 and 10 out of 24 (41.6%) in group 2 (p=0.120). There was no significant difference in the incidence of urinary leak, hematuria, or ureteric obstruction between the two groups (p=0.900). However, stent-related symptoms were significantly more in the delayed stent removal group (three in group 1 versus 18 in group 2) (p=0.001).

Conclusion: Prolonged stenting is associated with an increased risk of UTI and stent-related symptoms in immunosuppressed renal transplant recipients. The early removal of double J stents can be done in carefully selected patients to reduce stent-related complications in DDRT.

## Introduction

Renal transplantation is a cost-effective and desired modality of renal replacement therapy for patients with end-stage renal disease (ESRD), as it ensures a better quality of life [1]. Globally, there is an increase in the number of patients with ESRD. The most serious drawback in transplantation is organ shortage, which highlights the importance of encouraging deceased donor renal transplantation (DDRT). In recent times, DDRT is becoming increasingly common in India due to a shortage of voluntary, living-related organ donors [2-4]. Though deceased donor renal grafts have a higher risk of delayed graft function (DGF), the final graft outcome in successful cases is comparable to live-related donor grafts [5]. Ureteric anastomotic leaks are one of the major complications following renal transplantation. Ureteric double J stents have been routinely used by urologists to prevent such complications [6]. In our institute, we routinely place ureteric stents for all deceased donor renal transplants and remove them at four to six weeks after deceased donor renal transplant surgery. However, such delayed removal of stents can cause an increased risk of infection in both the early and late postoperative periods in susceptible patients. Early stent removal by the second week may logically reduce both urological and stent-related complications. Indian studies comparing early versus delayed removal of stents in DDRT are less. Hence, we conducted a prospective study comparing early and delayed double J stent removals in deceased donor renal transplant recipients.

## Materials and methods

The aim of this study was to compare urological outcomes and complications following early versus late removal of ureteric stents in DDRT. It was a prospective comparative study conducted in the department of urology and renal transplantation, Government Stanley Medical College And Hospital, Chennai, Tamil Nadu, India.

Our institute is a tertiary care hospital where over 120 DDRTs and 700 live-related donor renal transplantations have been performed since 1986. The study was approved by the institutional ethical committee. All patients above 18 years of age who underwent DDRT in our institute from August 2010 to December 2016 were included in the study. Patients with a past history of urinary tract infection (UTI), patients with an abnormal urinary tract anatomy like vesicoureteric reflux, posterior urethral valve, and urethral stricture were excluded from the study. A total of 80 patients were included after obtaining informed written consent. They were reviewed on the 14th postoperative day and were excluded from the study if they had any one of the following “high-risk” features:

1. Leak from the ureteroneocystostomy, diagnosed by a urine leak from the main wound or increasing drain output (fluid confirmed to be urine by a biochemical analysis)

2. Graft kidney showing moderate to gross hydronephrosis on ultrasonogram

3. Increased resistive index (RI) on Doppler scan of graft vessels

4. Impaired graft function, which can be due to DGF or rejection; DGF is defined as a graft dysfunction requiring dialysis in the first postoperative week; graft rejection is diagnosed by a biopsy of the graft

5. Persistent high drain output

6. Lymphocele confirmed by a graft ultrasound and a fluid biochemical analysis

**Figure 1:**
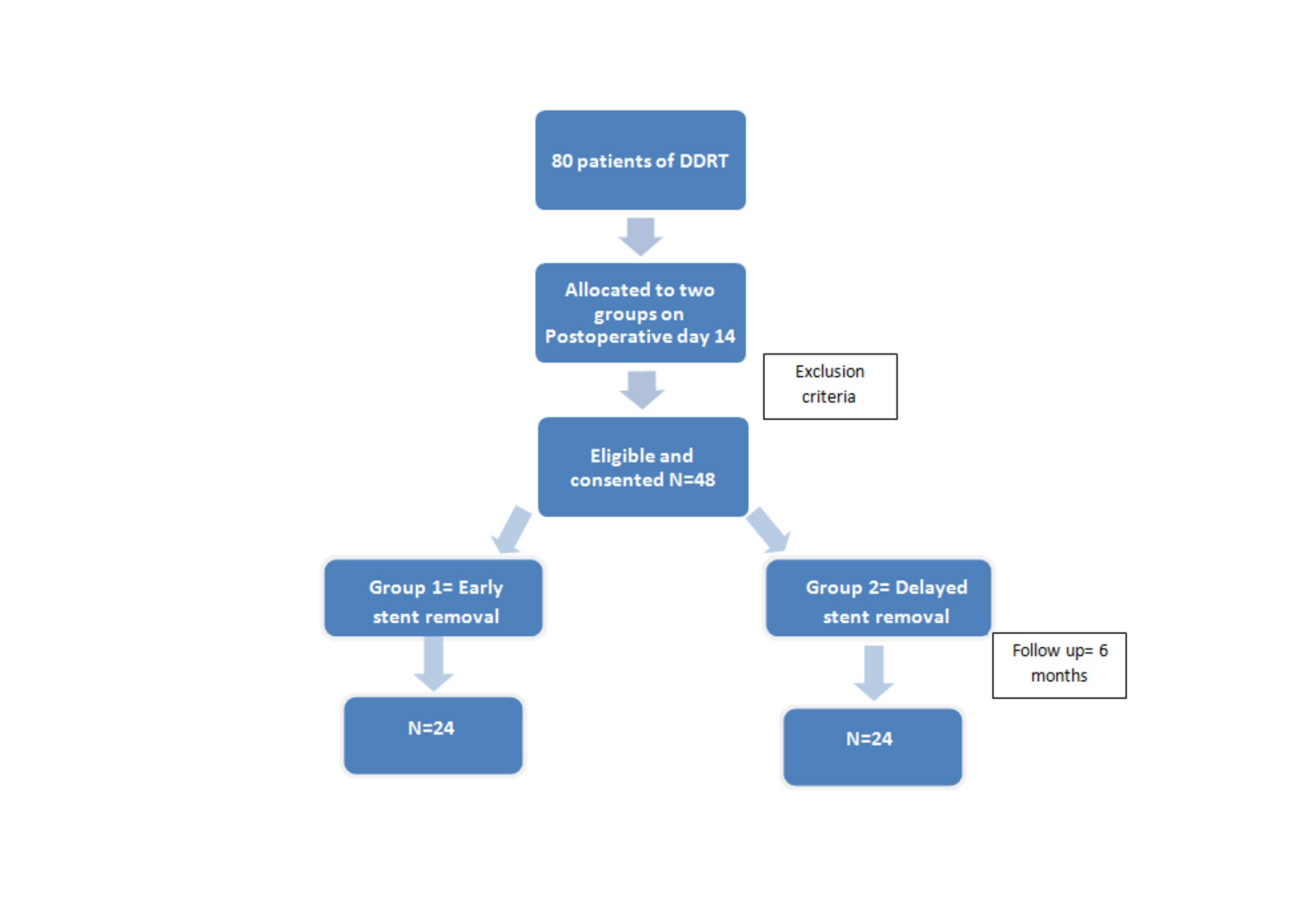
Flowchart of the study design N = number DDRT = deceased donor renal transplantation Group 1: stent removal on 14th postoperative day Group 2: stent removal in the sixth postoperative week

Thirty-two patients who did not meet the inclusion criteria were excluded from the study. The 48 patients selected were alternatively assigned to either group 1 (early stent removal) or group 2 (delayed stent removal) (Figure 1). In group 1, the double J stent was removed on the 14th postoperative day, whereas in group 2, the stent was removed at the end of six weeks. The stent was removed using a rigid cystoscope (Karl Storz GmbH, Tuttlingen, German, 20Fr) under local anesthesia and ceftriaxone 1 gram was injected intravenously 30 minutes before the procedure. The patients were followed up for six months. Imaging and urine examination with culture was performed whenever there was suspicion of UTI during the follow-up period of six months.

Deceased donor kidney procurement was performed by the same transplant team performing the recipient surgery and utmost care was taken to preserve the periureteric tissue and the “golden triangle” bounded by the gonadal vein, renal hilum, and lower pole of the kidney. The kidney harvested was immediately perfused with cold histidine-tryptophan-ketoglutarate (HTK) solution and transported immediately to ensure short cold ischemia time. The recipient surgery was started after a bench dissection of the harvested kidney. All procedures were done by a team of urologists with more than 15 years experience. After the venous and arterial anastomoses were performed, the ureter was spatulated and anastomosed to the dome of the bladder using running 4-0 vicryl to create wide, tension-free, refluxing ureteroneocystostomy. All patients received 4Fr/16 cm polyurethane double J stents. A 20 Fr Foley catheter was inserted intraoperatively and removed on the third postoperative day, after collecting a urine specimen for culture. Non-suction drains were used in all the patients and were removed when the drain output was less than 50 ml/day for three consecutive days.

All patients received intravenous prophylactic antibiotic (injection ceftriaxone 1 gm one hour before the procedure), which was continued postoperatively. The induction of immunosuppression was with anti-thymocyte globulin infusion commenced during the start of recipient surgery. All patients received a triple immunosuppressive maintenance regimen using tacrolimus, mycophenolate mofetil, and prednisolone. The serum levels of tacrolimus were checked on the fourth day after transplantation and on follow-up.

All patients were followed for the first six months and any urological complication during this period was documented. Urine microscopy and cultures were performed for all patients at one week, four weeks, three months, six months, and if there was fever or symptoms suggestive of UTI. The presence of two out of the following three criteria in a patient was diagnostic of UTI:

1. Fever (more than 99°F)

2. Positive urine culture (more than 105 colony-forming unit (CFU) per ml urine)

3. Symptoms of UTI (dysuria, suprapubic or graft site pain) [7]

Ultrasonogram of the graft with Doppler was done on the fifth day followed by four weeks, three months, six months after transplant, and whenever serum creatinine was abnormal.

The two groups were compared using the Chi-square test and the p-value was calculated. The mean values were compared using the Student's t-test. A statistical analysis was carried out at 5% level of significance and p-value <0.05 was considered statistically significant. The data were analyzed statistically using SPSS software (2008, Version 17.0, SPSS Inc., Chicago, US).

## Results

The demographic profile and baseline characteristics of the two groups are similar, making them comparable (Table 1). There was no statistical difference in the warm and cold ischemia times between the two groups (mean of four hours in group 1 and four hours 40 minutes in group 2).

**Table 1:** Demographic profile and baseline characteristics of groups 1 and 2 in the study Group 1: stent removal on the 14th postoperative day Group 2: stent removal in the sixth postoperative week ^*^Student’s t-test ^#^ Chi-square test N = number

Parameter	Group 1 N=24	Group 2 N=24	p-value
Mean recipient age	30.8±8 years	31.2±8.3 years	0.865^*^
Gender (N)			
Males	14	16	0.568^#^
Females	10	8	
Mean donor age	35± 8 years	32±10 years	0.291^*^
Mean donor serum creatinine (mg/dl)	1.6±0.3	1.7±0.4	0.175^*^
Ischemia time	240 ±40 minutes	280±30 minutes	0.175^*^
Mean serum tacrolimus levels on postoperative day-4 (ng/ml)	6.5±3.2	5.1±2.6	0.326^*^

**Figure 2:**
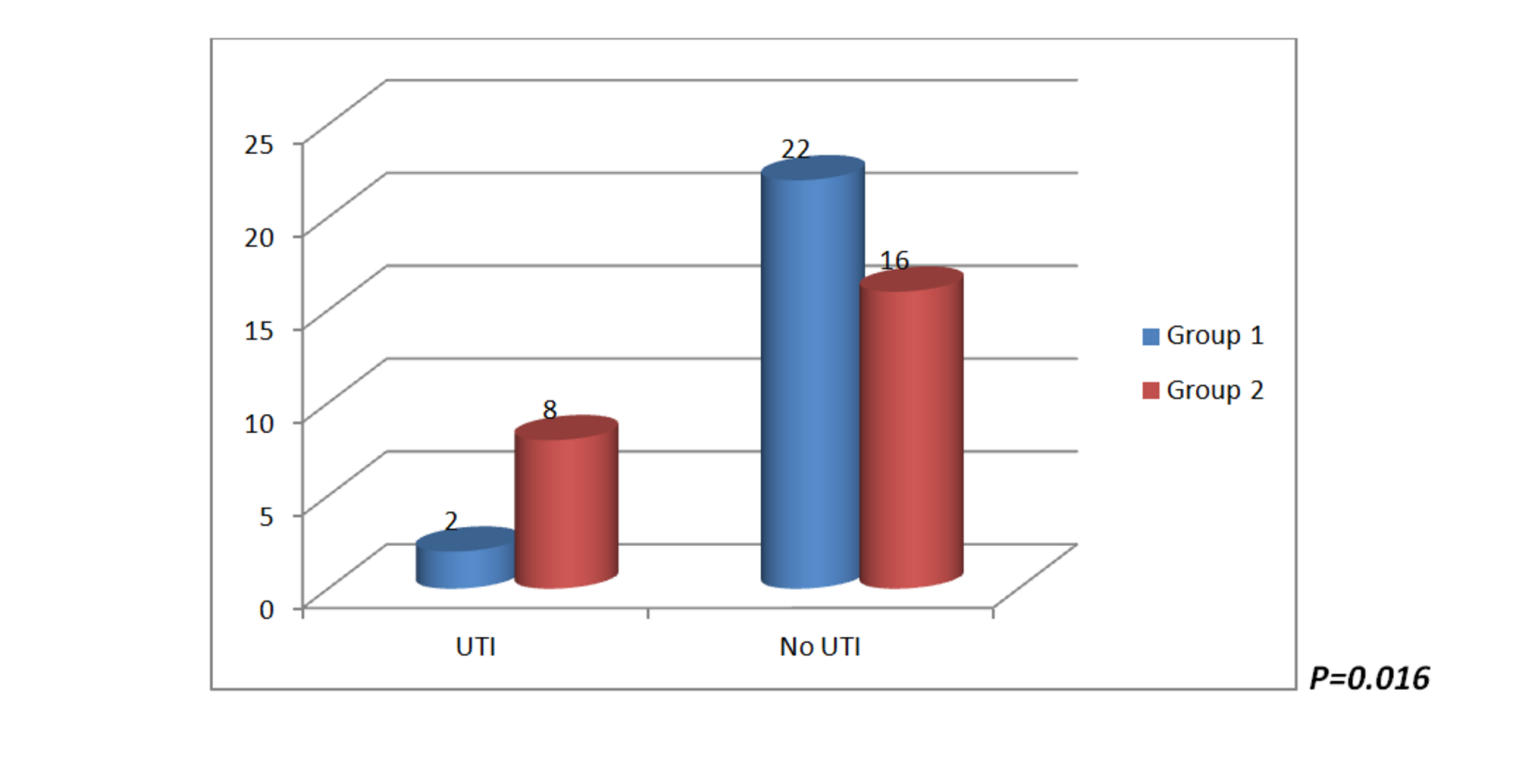
Incidence of UTI in group 1 and group 2 in the study UTI = urinary tract infection Group 1: stent removal on the 14th post-operative day Group 2: stent removal in the sixth post-operative week

**Figure 3:**
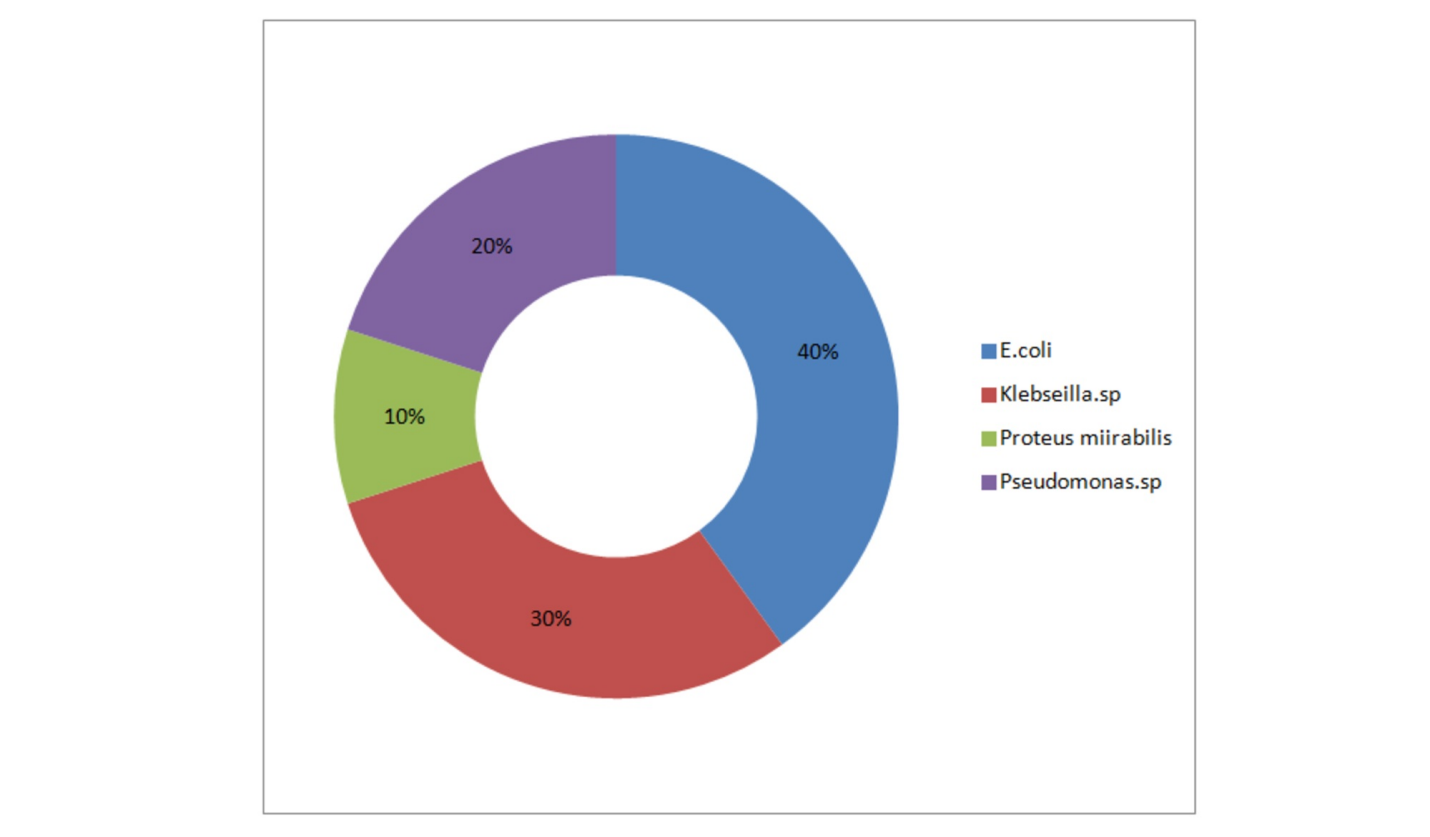
Bacteriological profile of urinary tract infection in patients included in the study

Six patients out of 24 (25%) in group 1 and 10 out of 24 (41.6%) in group 2 (p=0.120) developed asymptomatic bacteriuria. Two patients (8.3%) in group 1 (early stent removal) and eight patients (30%) in group 2 (delayed stent removal) developed symptomatic UTI (p=0.016) within the follow-up period of six months (Figure 2). Graft pyelonephritis was more common in group 2, with a single mortality, but it was not statistically significant. Escherichia coli was the commonest cause of UTI in both the groups (Figure 3). However, the bacteriological profile of UTI between both the groups was not statistically significant.

One patient in each group had a minimal ureteric leak following the removal of the stent and was managed successfully by percutaneous nephrostomy. Stent-related complications like stent migration, breakage, stent-related obstruction, or hematuria were not seen in either of the groups. Stent-related symptoms (dysuria, frequency) were predominantly seen in the late stent removal group (3 (12.5%) in group 1 versus 18 (75%) in group 2) (p=0.001) (Table 2).

**Table 2:** Group-wise comparison of infection and urological complications Group 1- stent removal on fourteenth post-operative day Group 2-stent removal in sixth post-operative week ^# ^Chi-square test N= number

Complication	Group 1 N=24	Group 2 N=24	p-value
UTI	2	8	0.016^#^
Asymptomatic bacteriuria	6	10	0.120^#^
Urinary leak	1	1	0.900^#^
Graft pyelonephritis	1	4	0.198^#^
Stent-related symptoms	3	18	0.001^#^

## Discussion

Our study is a hospital-based prospective comparative study to compare the early versus delayed removal of double J stents in DDRT. It included 48 recipients divided into two groups of 24 each. The incidence of UTI and stent-related symptoms were significantly more in the delayed stent removal group. Major urological complications were not statistically different between the two groups.

Double J stents have been extensively employed to reduce ureteric complications in renal transplantations throughout the world. Several meta-analyses in the past have proved the fact that ureteric stents reduce the risk of urologic complications [8-9]. However, the presence of stents is associated with a high risk of stent-related symptoms like dysuria, frequency, and hematuria [10-11]. It also promotes urinary tract infection (asymptomatic pyelonephritis, cystitis, and pyelonephritis) by the formation of a biofilm. Hence, recently, there has been a trend to avoid or reduce the duration of indwelling ureteric stents whenever possible. In a study by Dominguez et al., living donor transplantation, increased donor age, and short ureteric length were associated with increased ureteric complications, and the authors' advice stenting selectively in such cases instead of routine double J stenting of all ureters [12]. While this seems to be feasible in living donor renal transplantation, it cannot be directly applied to DDRT.

Though there are multiple studies comparing live-related renal transplantation with and without stents, studies are very few in DDRT [8,13]. Longer ischemia times, variable vascular anatomy, organ harvest techniques, incidences of rejection, and delayed graft function are the main factors that differentiate DDRT from live-related renal transplantation. Hence, the results of live-related donor renal transplantation studies cannot be extrapolated to DDRT. A recent study by Sarier et al. has shown that DDRT patients are more prone to stent-related UTI than live-related renal transplantation and the causative organisms are usually multidrug resistant in such instances [14]. This antibiotic resistance in organisms can be partly explained by the universally increased use of Pneumocystis jiroveci prophylaxis with sulfamethoxazole-trimethoprim (SMX/TMP).

Urological complications like ureteric leaks and ureteric strictures complicate 3%–9% of renal transplantations. These complications present early in the post-transplant period and have a significant impact on the morbidity and mortality of the patient. Double J stents have been unequivocally shown to reduce ureteric complications following transplantation [15]. In the present study, two patients (4.16%) had a ureteric leak, which was managed by percutaneous nephrostomy alone. Excluding the patients with risk factors for leaks from the study has probably caused the low incidence of leaks in our study. We did not have any patient with ureteric stenosis; however, the follow-up period was six months, which is a relatively short duration to assess stenosis rates.

The presence of an in-dwelling double J stent in the urinary tract of an immunosuppressed recipient promotes UTI due to several factors. Biofilm formation and colonization, stent-induced reflux, and immunosuppression are the main factors promoting UTI in such patients. Post-transplant UTI can induce graft dysfunction through free radical formation, cytokine release, cytomegalovirus (CMV) reactivation, and pyelonephritis-induced scarring. In the present study, 20.83% of the total of 48 patients developed significant UTI and 33.33% developed asymptomatic bacteriuria, with E. coli being the most common causative organism. In an Indian study by Khanna et al., 46.71% post-transplant patients with UTI had developed UTI while on antibiotic prophylaxis and E.coli was the most common organism [16]. Ranganathan et al. have pointed out that stents predispose the recipient to recurrent UTI even after the removal of the stent. UTI with the stent in situ is an important risk factor for recurrent UTI [17]. In our study, 75% of group 2 patients who developed UTI developed recurrent UTI. We excluded all patients with rejection, requiring additional immunosuppression, and, hence, the UTI rate is apparently low in our study.

Asymptomatic bacteriuria is significant in a transplant recipient and it must be treated, as it carries a risk of upper tract involvement and graft dysfunction by cytokine release [18-19]. In the present study, 33.33% of the patients overall had asymptomatic bacteriuria. The incidence was more in the delayed stent removal group though it was statistically not significant (p=0.12). A study by Fiorante et al. evaluated asymptomatic bacteriuria in renal transplant patients over a 36-month follow-up period. They found female gender and chronic glomerulonephritis as the etiology for chronic renal failure and double kidney transplant as the main risk factor for asymptomatic bacteriuria, and advice periodic surveillance for such groups of patients [20].

Double J stents are fraught with multiple complications like hematuria, dysuria, frequency, stent migration, breakage, encrustation, complications during endoscopic stent removal, and neglected stents compromising the whole renal unit [21]. UTIs are more common in stented patients and the risk can be mitigated by antibiotics. Stent encrustation and migration are more common in longer stents (20 cm) and longer periods of stenting (more than six weeks) [15]. In the present study, stent symptoms were six times more common in the delayed stent removal group, which is statistically significant. Dysuria was the commonest lower urinary tract symptom (LUTS) associated with stents. There was no incidence of a forgotten or neglected stent in the present study. This is in comparison to 5% reported in most other studies [11].

Studies comparing the infective complications of double J stenting in DDRT are few in the literature and, hence, we conducted this prospective study. Mathe et al. conducted a retrospective study on the DDRT of 310 patients who underwent DDRT with and without stenting. They reported that the risk of UTI is not increased by a double J stent. However, the high-risk criteria used in our study as exclusion criteria were not applied to patients in their study. Thus, the results were confounded by rejection and doses of immunosuppression. The urethral catheter was removed on postoperative days 4-7, which again increased catheter-associated UTI (CAUTI) in either group in their study [22]. In the present study, the urethral catheter was removed on the third postoperative day to reduce the confounding effect of CAUTI.

Ranganathan et al. conducted a retrospective analysis of UTI in 100 DDRT and live-related renal transplantations. The incidence of UTI was higher among patients with a stent compared to those without it (71% versus 39%). They concluded that stents increase the incidence of UTI. However, the study included both live-related and deceased donor renal transplantation [17].

In a randomized controlled trial by Parapiboon et al., 90 live-related and deceased donor renal transplant recipients were randomized to double J stent removal either on the 8th day or the 15th day post-transplant. The infective complications were analyzed. They concluded that early ureteric stent removal was beneficial only in live-related donor transplantation and the incidence of urologic complications was not different between the groups [23]. However, the DDRT patients in their study had a mean cold ischemia time of around 20 hours, higher donor creatinine, and prolonged periods of urethral catheterization than that in our study. Moreover, the rejection rate, which is a major risk factor for UTI, was not standardized between the groups.

Merits of our study

1. The study is a prospective study conducted on the Indian population.

2. The sample size is adequate (n=48).

3. Follow-up was for six months, which is longer than most other studies.

4. The study was done in the Indian setup, which predominantly includes illiterate people who are more prone to forgotten and neglected double J stents. Hence, early removal of stents during the hospital stay allays the anxiety of a forgotten stent.

5. Most of the recipients with high-risk factors were excluded from the study to assess the effect of stents on UTI.

Limitations of our study

1. It is a non-randomised study.

2. It is a single-institution study.

3. The follow-up is short, to assess the incidence of ureteric stenosis in the early stent removal group.

## Conclusions

The early removal of double J stents is safe in carefully selected patients undergoing deceased donor renal transplantation. It reduces the risk of UTI, graft pyelonephritis, and neglected stent, which is common in the Indian population. Based on the results of the present study, the authors recommend the early removal of double J stents, by two weeks, to reduce the risk of UTI. However, further multi-institutional studies involving a bigger number of DDRT recipients with longer follow-up are needed to ascertain the efficacy of such an approach in the Indian scenario.
